# The economic and social value of spa tourism: The case of balneotherapy in Maresme, Spain

**DOI:** 10.1371/journal.pone.0262428

**Published:** 2022-01-31

**Authors:** Jose Torres-Pruñonosa, Josep Maria Raya, Patricia Crespo-Sogas, Esther Mur-Gimeno

**Affiliations:** 1 Universidad Internacional de la Rioja (UNIR), Logroño, La Rioja, Spain; 2 Escola Superior de Ciències Socials i de l’Empresa Tecnocampus, Universitat Pompeu Fabra, Mataró, Spain; 3 Escola Superior de Ciències de la Salut Tecnocampus, Research Group in Attention to Chronicity and Innovation in Health (GRACIS), Universitat Pompeu Fabra, Mataró, Spain; Istanbul University Istanbul Faculty of Medicine: Istanbul Universitesi Istanbul Tip Fakultesi, TURKEY

## Abstract

The aim of this article is to assess both the economic and social value of balneotherapy and spa tourism, being the first paper in carrying out this analysis. The study has been conducted in Maresme, a region of Catalonia, Spain. On the one hand, an Input-Output (IO) model with a Social Accounting Matrix (SAM) has been carried out to assess the economic value. On the other hand, a Cost-Benefit Analysis (CBA) has been used to monetise the social value in this region, taking into account, among other concepts, direct and indirect health profits, given that balneotherapy helps to alleviate various diseases. The results show that whereas the economic multiplier is 1.529 considering the direct and indirect effects and 1.712 taking into account also the induced effects, which are similar to health and medical tourism multipliers, social value generates additional positive value, given that the cost-benefit ratio is 1.858. The theoretical implications of the paper as well as the findings’ implications for policy so as to encourage investments in spa tourism are discussed.

## Introduction

Health tourism is becoming one of the most important touristic activities in different countries [1–5], which has been defined by Goodrich & Goodrich [6] as an activity that aims to attract visitors so as to offer non-standard services, such as health care as well as appropriate equipment. Whereas Laws [7] defines health tourism as traveling to another destination from home in order to improve health as one kind of leisure, Lanz-Kaufmann & Muller [8] considers it as a sum of phenomena and relations that change people’s location and stay–away from their work places and permanent residence–to offer them health services that will restore mental, social and physical comfort. Finally, Carrera & Bridges [9] incorporates another objective apart from improving and restoring: health maintenance. Health tourism has a two-fold impact affecting both the economy [10] and society. Snyder [11] analyses the perception of medical tourism of different stakeholders, such as health care directors, physicians, tourism representatives, hotels and tourism operators, media and non-profit organisations, concluding that stakeholders connected to medical tourism expansion or tourism sector took a positive view, whereas those connected with public health were concerned about whether health tourism may generate inequalities within the country. In this sense, Butler & Szromek [12] recommend including not only customer’s value and the value captured by enterprises but also the value for the community–for instance, suppliers selection policy, natural environment protection, customers’ relationship in regard to pro-community behaviours involving the local community–within the value proposition of health tourism enterprises.

Weaver & Lawton [13] include visiting spa resorts in combination with leisure activities in the concept of health tourism. In this regard, some papers [2, 12, 14, 15] aim to know and understand spa business mechanisms, proposing a concrete and sustainable business model for this kind of health resort enterprises, highlighting the excessive pressure on tourism as well as the exploitation on natural resources as a negative impact for society.

Spas offer an alternative to conventional tourism, not only because of their intrinsic characteristics as health resorts, but also because of the possibilities they offer in the environments where they are located. Therefore, it is vital for these resorts to be able to combine perfectly these two activities of health and tourism, enriching them with new offers of leisure activities [16, 17]. Nonetheless, balneotherapy is indeed the greatest strength and the most differentiated feature of spa tourism.

The terminology used in international literature for the application of water for therapeutic purposes, varies according to its country of origin. This suggests difficulties in consensus and that it is important to take into account the country of origin in order to understand the terminology being used. For instance, in English-speaking countries, the term spa is used to refer to the establishments where mineral-medicinal and/or thermal waters are (mineral springs spas); conversely, the name “balneario” is used in Spain (thereinafter, spas will refer only to those where balneology is used, not including other kinds such as thalasso). Furthermore, there is a differentiation between the word balneotherapy, which refers to bathing, and the term crenotherapy, which includes other aspects such as inhalation, mud applications and drinking, in addition to bathing [18]. In any case, health resort medicine, whether we are talking about de balneotherapy, crenotherapy, thalassotherapy or hydrotherapy, has experienced a breakthrough in recent years, a progress towards evidence-base science, taking into account its contribution to the different ICF levels (the WHO International Classification of Functioning) [19]. Therefore, we can say that balneotherapyand crenotherapy are focused on the use of buoyancy, the physical and thermic properties, in addition to the effects of the water’s chemical elements on the organism. The effects produced by the mineral-medicinal water will vary depending on its most abundant chemical elements [20]. Traditionally, balneotherapy treatments are used in the prevention, treatment and rehabilitation of musculoskeletal and rheumatoid disorders [21]. Nonetheless, there are other applications, such as in dermatologic processes [22], chronic pain-based and immuno-inflammatory diseases [23, 24], neurologic diseases and even in psychiatric illnesses [25].

Therefore, spa tourism does not only generate economic value but also social value for their users and, at the end of the day, for society. Traditional accounting systems aim to identify the financial and economic value created by companies for shareholders [26]. Nonetheless, over the last decades, enterprises have also been regarded as generators of social value [27, 28]. Whereas economic value is oriented to the value created for shareholders, social value aims to quantify the value created for all stakeholders [29, 30]. Although the assessment of social value has a long tradition [31], its evaluation is not yet adequately standardised. There are different methodologies [32–34] to quantify social value, such as social accounting [35, 36], social value monetisation [37], Social Return on Investment (SROI) [38] or Cost Benefit Analysis (CBA) [39, 40]. Whereas firms have been commonly regarded just as mere generators of economic value, relegating the social value they might generate to a secondary role, some researchers have integrated both values into a more cohesive approach [41–50]. This is the approach that this paper has taken, aiming to calculate the economic value of spas by means of an input-output (IO) analysis as well as their social value by means of a CBA.

As far as tourism is concerned, most papers analyse only the economic impact of industries and events, mainly by means of IO analyses [51, 52], without taking into account the social value created [51–60]. For instance, Zhu [61] performs an economic analysis of the impact of medical tourism industry on Kwangwon province, South Korea, concluding that the multiplier is 1.53 and estimating that local financial self-sufficiency improves by 0.1603% annually. Eusébio et al. [10] carry out an IO analysis of the Portuguese Health Tourism Programme for the elderly concluding that this programme has high multiplier effects, originating outstanding economic impacts in terms of employment, output, value added and household income. Although some papers use CBA [62] to assess only the economic impact of tourism [58, 63, 64], in some tourism articles a CBA has been conducted in order to deal with social value creation [39, 65–68].

Spas have a high potential to attract visitors to destinations. Nevertheless, they have not received much attention in literature, especially with regard to quantifying both the benefits and costs of investing in spa/balneo centres as well as offering multipliers that can be used to assess and make investment decisions. There are many articles, though, that analyse the cost-effectiveness of spa therapies with regard to different diseases. Allard et al. [69] analyse the reimbursement services in the French health care system’s patients, after a spa therapy. The authors do not appreciate a decrease in the use of the health system by patients who had used spa therapy previously; nonetheless, in the case of those using it for the first time, a decrease in the demand for medical attention is observed. For this reason, the authors pose the need for a review of the French social security reimbursement system. In the study carried out by Fioravanti et al. [70], osteoarthritis patients undergoing a treatment cycle by means of balneology and mud packs during two consecutive years are monitored. Sulphuric, chloride, sulphate and bicarbonate waters are used. The authors show a decrease in the resources devoted to this group by the health system. The study carried out by van Tubergen et al. [71] analyses a three-weeks treatment in two spas located in the Netherlands for patients with ankylosing spondylitis. These treatments combine hydro-therapy with physical exercise. The study shows favourable cost-benefit and incremental cost utility ratios for this type of population when the standard treatment is combined with three-weeks treatments at the spa and exercise. In the case of other authors, such as Zijlstra et al. [72], specialists use thalassotherapy in combination with group exercise and training sessions to treat fibromyalgia patients, during two and a half weeks. An improvement in the patients’ quality of life is observed, while they analyse an increase in the incremental costs of this combination of therapies that requires further study to find out its profitability. Another study to take into account is the one conducted by Mourgues et al. [73], with post mama-breast and in treatment remission patients that took part in a two-week multi-modal program that combined balneotherapy with exercise and diet. They conclude that multi-modal treatment for this kind of patients is profitable one year after their stay, since patients improve their activities and participation taking into account ICF (International Classification of Functioning). When it comes to knee osteoarthritis, according to Ciani et al. [74], the application of 12 mud and mineral waters treatments during two weeks at the spa (mud-packs and hot mineral baths) improves the symptoms until 3–6 months after the treatment. Additionally, they show a decrease in the cost per patient over one year of monitoring; therefore, they conclude that mud along with medicinal mineral water can be a recommended therapy, complementary to conventional treatment. In the case of the Spanish population using the IMSERSO [*Instituto de Mayores y Servicios Sociales* (Institute for Older Persons and Social Services) program, a study was conducted in which is appreciated a reduction in the use of medicines and a positive impact in the patients’ perception of their quality of life after a 21-day spa treatment [75]. If we focus on chronical low back pain, according to Balogh et al. [76], the application of sulphuric waters during 15 consecutive days (with the exception of Sundays), seems to be more efficient than the same techniques using potable water (hydrotherapy). Furthermore, the authors stress the need for further research in balneology cost-profits. In the same line, the review conducted by Pittler et al. [77] highlights that the use of hydrotherapy and balneotherapyin the treatment of chronic low back pain has significantly beneficial effects when applied under the supervision of professional experts. Finally, the review conducted by Mennuni et al. [78] highlights that the combination of the mud therapy applied in spas, with the conventional treatment for knee osteoarthritis, can help to improve the patients’ functioning and alleviate their pain.

Whereas many are the papers that deal with cost-effectiveness of spa therapies, this is the first one that analyses the economic value of spa tourism by means of an IO analysis including a Social Accounting Matrix (SAM). Furthermore, this is the first article that takes into account social value by means of conducting a CBA of spa tourism. All in all, the main contribution to the field of this paper is the assessment of both the economic and social yield of investing on spa tourism. Given that this is the first paper that carries out an IO analysis in regard to spa tourism, we have searched for previous studies on medical and health tourism and the total multiplier ranges between 1.654 and 1.742 [10, 61, 79]. Therefore, the spas tourism multiplier from Maresme is not expected to be exactly among this range but it is expected to be somehow similar to these values.

After the introduction, section 2 sets the context of the analysis by means of discussing spa tourism and Maresme region. Section 3 analyses the methodologies used, namely IO analysis and CBA. Section 4 shows and discusses the results obtained. Finally, section 5 finishes with the main conclusions of the paper and suggests future lines of research.

## Context of the analysis: Spa tourism and the analysed region

The first spa/balneo centres were initially designed to recover health. Over 2000 years ago, in many cultures such as Arabic, Greek and Roman, water was used with therapeutic purposes and, for this reason, facilities were built on the outskirts of natural fountains, from which medicinal waters flow, in order to be able to use them to treat several diseases [80].

The tradition of thermal baths has been handed down from one generation to the next. Throughout the first half of the 20^th^ Century spas were viewed as places where people would go only when they needed therapeutic stays and medical prescription. In the 1980’s, hydrotherapy underwent a revival and spas reached their maximum splendour, thanks to the influence of several organizations, administrations and health specialists, who raised awareness of the fact that spas are a favourable environment, not only to cure diseases, but also to prevent them and to gain a better quality of life from an integral perspective [81].

Spas have evolved from health-care establishments in the 19^th^ Century, to leisure and tourism facilities in the 20th Century. So, after having been in decline for some years, spas have nowadays become centres of high-quality tourist attraction, since they are becoming leisure areas, where a wide range of tourism and leisure activities, other than baths in mineral-medicinal waters and massages are offered (without losing their raison d’être, namely the use of mineral-medicinal waters). Catalonia [Nomenclature des Unités Territoriales Statistiques II (NUTS-II)] has not remained oblivious to this fact and is among the most important European thermal centres because of the properties of the mineral-medicinal waters that can be found at the several spas located in this region.

This paper will focus on two thermal villages located in the Maresme county (NUTS-IV): Caldes d’Estrac (NUTS-V) and Arenys de Mar (NUTS-V), municipalities for which health tourism is an important source of resources. (Given that our paper focuses on balneotherapy, a third spa that is located in Maresme region–Hotel Colón Thalasso Termal–has not been included in the study, because it is a thalasso). Table 1 shows their main features.

**Table 1:** Comparison between spas.

Location	Caldes d’Estrac	Arenys de Mar
**Name**	Balneari Públic de Caldes	Balneari Titus
**Opening year**	1818	1794
**Number of visitors per year**	2,100	1,220
**Rooms**	No	No
**Management**	Public	Private

Caldes d’Estrac is a very important thermal village. Since the days of ancient Rome (when the place was named Aquae Calidae), its thermal baths have been the main attraction of the village. The properties and benefits of its chloride water have always been highly appreciated [82]. It contains cations (all the measures are expressed in % mEq/L): sodium (80.713), potassium (2.529), magnesium (2.951) and calcium (13.807) / anions: bicarbonates (20.013), chloride (63.190), bromides (0.071), carbonates (3.046) and nitrates (0.952). It is a low-mineralised water and it is high-brine. It is a very soft water (under 100 mg/L of CaCO3), rising to the surface at a temperature of 38.8°C. It is a hyper-thermal water. It is an alkaline water with a pH of 7.37. Due to its chemical composition this water is stimulant, anti-inflammatory and diuretic. Being a thermal water, it affects blood circulation, improving its sedative and relaxing effect. Very indicated in chronical disorders of the neuro-musculoskeletal system. It is used in bronchial spams, rhinitis, chronical laryngitis, nasal congestion and bronchitis as well as in dermatitis and gynaecologic processes. Drinking it, once cold, improves uric acid, oxalic calculations and inflammation of the urinary tract. It has effect on constipation. The public spa dates from 1818.

The seaside town Arenys de Mar is another important town that has purely mineral-medicinal waters. which were also discovered during the Roman era. In the year 1794, the thermal waters named Banys de Can Titus were opened to the public. After a short period of inactivity, Can Titus reopened its doors in 1992, renovated as a modern spa. Balneari Titus has water sodium chloride and radioactive water (radon) [83]. It contains cations: calcium (15.0 mg/l) and silica (52.3 mg/l) and anions: chloride (280.4mg/l), bicarbonate (158.7 mg/l), magnesium (0.6 mg/l), sulphates (53.0mg/l), fluorides (7.2 mg/l), bromides (1.0 mg/l), sodium (259.4 mg/l), potassium (7.6 mg/l), Chloride (280.4 mg/l) and Lithium (0.54 mg/l). It is also a low-mineralised water and it is too high-brine. It is a soft water (between 100 and 200 mg/L of CaCO3) and mesothermal. It comes out at a temperature of 37°C. This water is alkaline with a pH of 7.92. It is sedative, relaxing, anti-inflammatory and disinfectant. Very indicated for rheumatology and post-traumatism, as well as for non-productive respiratory processes (Asthma, Bronchial Hyper-reactivity). It acts as stimulator on mucosal membranes. Drinking it (hydroponic cure) facilitates digestion and improves constipation.

## Methodology

The economic value of spa tourism will be assessed by means of an IO analysis and the use of Social Accounting Matrix (SAM). The direct and indirect economic benefits will be computed by means an IO analysis. Direct effects are the increase in companies’ sales revenues or tourist spending which was determined by means of a survey as the main input to be estimated. Thereafter, firms need other firms located in the county to provide them with inputs; these other firms, in turn need, additional firms to provide them with inputs, and so on. These are the indirect effects that normally affect many sectors of the economy. The result obtained is the multiplier effect, which is the increase in the final income generated by the chain reaction triggered in the local economy when there is new extra spending in this category. Finally, the economic value analysis will be complemented by a SAM to calculate the induced effects [84–86]. We refer to induced effects when anyone whose income increases (such as employees) spends the new income generated by the visitors’ spending in the region. A SAM is an economy’s snapshot for a specific year and it consists of a double-entry table that describes and synthesizes economy’s structure in terms of the connections between production, demand and the distribution of income. Additionally, it includes the expenditure and revenue of all the institutions and agents of an economy [85].

The social value of spa tourism will be assessed by means of a CBA that analyses, in addition to economic and non-economic costs, the economic benefits as well as other additional benefits derived from investments in spa tourism. Therefore, this methodology goes one step beyond because it assesses several social variables and some effects that IO analysis does not take into account. CBA includes costs and benefits that are inherently social, whether tangible and intangible. Cost-benefit ratio will be the final output of the CBA model, which is a single measure of social yield or return, representing the number of times benefits are higher than costs. If this ratio is higher than 1, social benefits are higher than costs, proving that the investment is socially profitable. By means of this ratio it is possible to compare alternative uses of funds and resources, and consequently decision-makers are able to compare the social value of different projects when taking investment decisions. In the public sector, CBA is frequently used to make investment decisions. Actually, many public projects need to pass a CBA prior to their implementation in order to quantify and demonstrate their net social value.

### Economic impact of spa tourism on the Maresme county: IO analysis and SAM

The money spent (on restaurants, food, hotels, shopping, and so forth) in Maresme by visitors attracted by spa tourism generates an economic impact. A traditional IO model has been chosen to conduct the analysis of the economic impact (the total amount of direct and indirect effects) generated by investing in spa tourism in Maresme, given that IO economic impact models are frequently used in studies on tourism and recreation [87]. In IO models, inter-industry IO relations as well as final demand (such as investment, consumption, imports, exports) are taken simultaneously into account [84]. Therefore, the impact caused by an external demand shock upon the economy as a whole can be estimated (the expenses of tourists–day-visitors and visitors who stay overnight at the destination). Tourism industry encompasses several sectors and, consequently, any supply-side shock and/or demand given to this sector brings about not only industrial but also inter-industry impacts. As stated earlier, with regard to the spatial dimension of these effects, they are limited to Maresme county.

To analyse the economic impact, it is necessary to use IO tables. This method allows quantifying the impact of a change in the demand or in the activity of a productive sector upon the economy as a whole. More precisely, the IO table allows calculating the economic effects and, at the same time, distinguishing the consequences produced in each productive sector [88, 89], which helps to understand sectorial interrelations. These relations are represented by a matrix in which are compiled the goods and services flow values of the economy within a specific territory. The table presents two readings: on a horizontal axis, the rows record the different uses of each sector’s production, which can be either intermediaries or final users; on the vertical axis, columns reflect, for each sector, the resources that have been used to obtain the effective production.

All things considered, IO tables are useful for two reasons: firstly, because they provide a representation of the economy as a whole divided into activity branches by means of quantifying the transactions among them, the production of each branch destined for final demand and their use of primary resources. Secondly, it allows analysing the effects produced when there are changes in the demand of any branch over the rest of them.

The method that will be used in this paper will be explained in the following lines. Using the information included in the IO table, we can obtain a set of elements that make up the calculation model used to carry out the analysis of impact and the multiplier effects of an investment in a specific territory. The model to quantify the economic impact is based on the technical coefficients matrix and on the Leontief inverse matrix. The vertical technical coefficients are the translation into unitary values of the data included in the columns of the IO table. So, they define the consumption needs as intermediaries of each productive branch in order to obtain one product unit. The formula is the following:

aii=xij/xj (1)

Where: *xij* is the branch’s amount of products *i* used by branch *j* to obtain its production *xj*. To put it another way, it is the need that industry *j* has of products provided by industry *i* to obtain one unity of the produced good. Consequently, the set of technical coefficients for each productive branch defines what is known as the intermediate consumptions matrix (*a*).

The coefficient technical matrix allows analysing the effects derived from the modifications on the economic activity. Nevertheless, these effects are produced beyond the productive industry whose activity has increased, since increasing in one unit the final demand of products of branch *j* will imply the supply of all the intermediate inputs needed to carry out the production, although it will also trigger a chain of subsequent needs. This means that increasing the activity of one productive branch will generate an increase in the demand of inputs to be able to carry out this activity:

$$X_{1}=A\cdot D$$ (2)

*A* is the technical coefficients matrix, *D* the vector of the increase in demand and *X*_*1*_ the needs for provision of the new inputs. But this increase in the need for inputs brings about a new need for inputs to produce them:

$$X_{2}=A\cdot X_{1}=A\cdot (A\cdot D)=A\cdot D$$ (3)

And so on and so forth, because each new production requires new inputs to be supplied. This interactive model allows to grasp the sequential chain of needs for inputs to be able to meet the needs for inputs of the productive system. The result of this chain takes the following form:

$$X=D+A_{1}\cdot D+A_{2}\cdot D+A_{3}\cdot D+A_{4}\cdot D+\ldots =[I+A+A_{2}+A_{3}+A_{4}+\ldots ]\cdot D$$ (4)

$$X={\left[I-A\right]}^{-1}\cdot D$$ (5)

Where *I* is the identity matrix, *[I − A]*^*-1*^ is named Leontief inverse matrix or demand multiplier, which reflects the needs for supply of inputs that result from a modification in the activity of one or several productive branches. Therefore, the sum of the elements of each of the Leontief inverse matrix columns expresses, as stated earlier, the production increase generated in the economy as a whole due to the activity generated by a productive sector, including the initial activity from which the whole effect is derived.

Finally, to estimate the induced effects of spa tourism, the Catalan IO table was inserted into a SAM [90]. By means of this procedure, the relationship between spa tourism industry and macro-economy was obtained.

### Questionnaire data

A survey was administered to calculate the direct economic impact. The fieldwork was conducted during the months of March, April and May, 2016 in two spas located in the Maresme county: the spa *Balneari Titus* in Arenys de Mar and *Balneari Públic de Caldes d’Estrac*. The questionnaire consisted of 22 questions divided into 5 differentiated sections: socio-economic data, visitor/tourist’s profile, visit’s profile, level of spending and a final section named “willingness to spend”, which purpose is to make an assessment of spa tourism. The population is 3,320 spa visitors per year (visitors are considered different individuals regardless of their number of visits; so, a single person could visit the spa several times in a year, being counted just as a single visitor); of which 1,220 corresponded to the spa Titus and 2,100 to the *Balneari Públic de Caldes d’Estrac*. In total, 305 surveys were conducted, obtaining verbal consent from participants once they were informed. Keeping into account the total population, it is estimated with 95% confidence that, with this sample’s size, the maximum error rate is 4.82%. During the survey process, surveys have been administered to different individuals randomly selected from among the two spas’ visitors. (Randomisation was achieved by using systematic random sampling which is a very common technique in which every *k’th* element is sampled. In this case, we surveyed every person that visited the spa. In other words, a sampling frame was taken and the size of the frame, *N*, is divided into the desired sample size, *n*, to get the index number, *k*. Then, every *k’th* element in the frame is chosen to create the sample.) Furthermore, the days have been scheduled by the managers of the centres, choosing those with highest attendance of visitors.

### Cost-benefit analysis

With the aim of obtaining a final single figure of net social value–although different partial results can be obtained in regard to different social benefits–, CBA has to assess, in monetary terms, all costs and benefits (present time equivalent). Among these costs and benefits are included those with no market prices (intangibles; [91]). It was easier to calculate benefits obtained from the economic impacts and the spending of visitors (for example, there is a market price to stay in paid accommodations, for food and beverages or sports apparel). The market price expresses, under certain circumstances (for instance, in perfect competitive market), the individual’s valuation on goods or services to measure the extent to which they are willing to pay for them. In regard to intangibles, market price or observable monetary figure for the valuations of individuals do not exist. Hence, different methodologies need to be used to value intangibles. The basic methods are revealed preferences techniques (or indirect methods) and stated preference techniques, such as contingent valuation methods (or direct methods). Revealed preferences techniques are based upon the market decisions of an individual (for instance, individuals who pay or accept a compensation when purchasing or selling). These decisions can be used to “reveal” the valuation individuals place on intangibles. Some of the most frequently used techniques, which have been used in this study, are hedonic prices, the travel cost method, productivity models and human capital models. A specific method is required for each case of analysis. For instance, to assess the Direct Health Benefits, Eq (6) will be used:

$$DHB=\sum_{d=1}^{n}I_{d}{\cdot C}_{d}\cdot P_{d}\cdot V$$ (6)

where *DHB* is the Direct Health Benefits, *d* is the different diseases improved by balneotherapy, *I*_*d*_ is the percentage of improvement due to balneotherapy, *C*_*d*_ is the direct annual cost for the Spanish National Health, *P*_*d*_ is the percentage of the Spanish population suffering from these diseases and *V* is the total number of annual visitors that use Maresme spas during 21 or more days consecutively [92].

In this regard, Llor [93] states that there is scientific evidence that the spas’ mineral-medicinal waters, from now on balneotherapy, alleviate the pain in some illnesses. Llor [93] uses the US Agency for Health Care Policy and Research system to rate the strength of scientific evidence [94], where the following six levels are used and the recommendation grade is given: Ia. Meta-analysis of high-quality clinical studies (A); Ib. At least one high-quality clinical study (A); IIa. Well designed nonrandomized prospective study (B); IIb. Well designed quasi-experimental study (B); III. Well designed observational studies (B); IV. Documents or opinions from think tanks and/or clinical experiences from prestigious authorities (C). In regard to the recommendation grades provided in parentheses, A means high, B means medium and C means low. In this article we are going to focus just on grade A diseases, which are the ones with several corroborated good-quality clinical studies, or at least one good-quality clinical study of scientific evidence, respectively. So, the analysed diseases are Arthrosis, Rheumatoid Arthritis, Fibromyalgia and Low Back Pain.

## Results

### Direct economic impact

The results of the sample were contrasted with the results of the population (namely, all the users of both spas whose profile was provided by the spa’s managers) and both groups share the same characteristics in regard to gender, age, motivation, residence, percentage of IMSERSO users, as well as for the rest of the analysed features. Therefore, we can consider that the sample–which is statistically significant and was selected randomly–is representative of the heterogeneous population. The results of the survey in regard to spa visitors’ profile are shown in Table 2.

**Table 2:** Summary of the results obtained from the survey. Visitors’ profile, their habits and other relevant information.

Age	>61
Women	66%
University graduates	48%
Retired	50%
Origin: Catalonia	83%
Frequency of sporadic visit	75%
Relax as main motivation	50%
Visitors accompanied by partners	50%
Visit typology: spend night in the county (tourists)	60%
Type of accommodation: 4-star hotel	77%
Type of accommodation: Full-board	72%

The average age is in the region of 57 years, although the predominant age group is over 61 years old. In all age ranges, there is a majority of women. With regard to the origin, 83% of the respondents are from Catalonia, being Barcelona province (NUTS-III) the origin of the majority of users. 15% come from the rest of Spain and 2% are international tourists, basically from Andorra, France, United Kingdom and Russia. In regard to education, the majority of spa users are people with advanced education. Specifically, 48% of the respondents took a university degrees and 27% finished their post-compulsory secondary education. As far as employment status, most spa visitors are retired people (50%), which explains why the predominant age range is over 61 years old. Most spa potential customers use IMSERSO programmes. (IMSERSO is a Spanish public institution that promotes tourism and hydrotherapy programmes among the elderly, which are cheaper than the market price, given that they are partly paid by this institute). Additionally, 41% of visitors are active people, whereas 6% are unemployed and 3% are students. This small percentage can be explained by the students’ purchasing power, which does not allow them to afford this kind of services and because spa tourism is less appealing for the young, who prefer other kinds of tourism. By means of these data, the user profile of spa tourism can be synthesized as women over 61 years old and consequently, retired but with university degrees, having a medium-high purchasing power.

With regard to the frequency of spas visits, 75.41% of respondents go to spas sporadically (in most cases once or twice a year), 10.49% visited a spa for the first time, 8.20% monthly and 5.90% weekly (5.90%). Their main motivation to visit the spa is relaxation (50.16%) and recovery (28.20%), followed by healing a sickness (13.77%), prevent sicknesses (5.90%) and leisure (1.97%). On the other hand, 50% of users are accompanied by their partners, whereas 15.41% are accompanied by their families and 2.30% do it in groups. It is noteworthy that 3 out of each 10 users go to the spa alone.

The behaviour of spa users with regard to their accommodation consumption habits is analysed. It is essential to differentiate between day visitors–who do not stay over–(40%) and tourists–who do it (60%). With regard to the accommodation typology, the most commonly chosen by visitors are 4-star hotels. This analysis of the accommodation typology should be accompanied by an analysis of the type of stay. In the questionnaire there are 4 possible types of stay: self-catering, bed and breakfast, half board and full board. Nonetheless, another possible type of stay was added, named private accommodation, in order to be able to include those staying in apartments, second homes, friends and relatives houses and others; since these accommodations have their own kitchen, those who use it will spend less in eating and drinking than those who will use restaurants. The predominant stay typology is full-board. This type of stay is linked to the type of accommodation most commonly chosen by visitors: 4-star hotels. Out of 60% visitors who need accommodation, 72.7% are tourists on a package tour of the IMSERSO social hydrotherapy program. Since none of the two spas offer accommodation, they have an agreement with the 4-star Hotel Volga, located in Calella, to offer accommodation.

The analysis of the visitors’ expenditure in the Maresme county during their visit has been divided into three different groups: accommodation expenditure and treatments (this is so because visitors in the IMSERSO group cannot provide a breakdown or their expenses in accommodation and treatments, therefore, the average spending in both concepts together was estimated), spending in restaurants and provisions and, finally, other expenses, which include shopping, culture and sports.

#### Expenditure in accommodation and treatments

The total average of the visitors’ expenditure in accommodation and treatments in each of the spas was calculated (being €359.53 for the spa in Caldes d’Estrac and €136.27 for the spa in Arenys de Mar) and it was multiplied by the number of visitors per year of both spas (1,220 and 2,100 respectively). As a result, the total expenditure in accommodation and treatments amounts to **€724,427.60.**

#### Expenditure in restaurants and provisions

Following the same procedure, the total average expenditure in restaurants and provisions was calculated: €74.69 for the spa in Caldes d’Estrac and €49.19 for the spa in Arenys de Mar. The total expenditure of users in restaurants and provisions amounts to **€194,420.80.**

#### Other expenditures

The visitors’ expenditure in shopping, culture and sports has been estimated using the same method, namely calculating the users’ total average expenditure in these three concepts in both Arenys de Mar (€88.98) and Caldes d’Estrac (€24.75) and it has been multiplied by the number of users per year. The total expenditure in shopping, culture and sports amounts to **€160,530.60**.

Therefore, taking into account the previous results, the total direct expenditure of spa users in Maresme amounts to **€1,079,379.00.**

It has also been analysed what is the surplus value of spas customers. Firstly, the questionnaire asks whether they would keep coming even though the expenses increase in certain quantities. If the answer is yes, they are asked how many more euros would they be willing to pay, offering the following options: from €30 up to €210. We can see that 63.61% of users claim that, even if the cost is increased, they would go to spas, whereas the remaining 36% would not pay more money. Furthermore, bearing in mind that 63.61% of users would increase their expenses, it is analysed the amount; that is to say, how many more euros would they be willing to pay. 66.49% of users would be eager to pay up to 30 more euros. Table 3 shows the rest of results. To calculate how much more, on average, each person would be willing to pay, the sum of multiplying the extra amount by the percentage is calculated, as shown in Table 3.

**Table 3:** Willingness to pay calculation (in euros).

Extra amount (in euros)	Percentage	Result (in euros)
€30	66.49%	19.95
€60	21.65%	12.99
€90	7.73%	6.96
€120	3.09%	3.71
€150	.52%	.77
€180	.00%	.00
€210	.52%	1.09
**Total**		**45.47**

The result of multiplying the extra amount they would be willing to pay by their percentage, is multiplied by the 194 people who claim to be willing to pay more money, and divided by the size of the sample (305), resulting in that each person would be willing to pay on average €28.92 more.

### IO analysis

The visitors’ total average expenditure in accommodation, treatments, restaurants, provisions, shopping, culture and sports has been estimated in the previous section and amounts to **€1,079,655.98** which is considered the total direct expense. This figure allows us to estimate the indirect impact, by assigning it to its corresponding sector in the IO table (to 10 sectors) of Catalan economy, which has been assigned to sector 4 (commercial services, transportation and hotel industry). Additionally, the expenses of the two spas (see Table 4) have been assigned to their corresponding sector. Thus, staff and marketing has been assigned to sector 8 (professional, scientific, administration and auxiliary services), the electricity, materials, gas and maintenance to sector 2 (industrial products and sanitation), municipal business, water tax and taxes are assigned to sector 9 (public administration services, education and health care), hotel’s expenses, van and diesel oil as well as miscellaneous have been assigned to sector 4 (commercial services, transportation and hotel industry) and, finally, the expenses in the new building to sector 3 (building works).

**Table 4:** Annual costs of spas (in euros).

	Balneari Públic de Caldes (Caldes d’Estrac)	Balneari Titus (Arenys de Mar)	Both
Staff	96,000	112,700	208,700
Electricity	12,000	9,500	21,500
Equipment	0	22,000	22,000
Gas	3,000	9,000	12,000
Municipal Tax	15,000	0	15,000
Catalonian Taxes	2,000	0	2,000
Marketing	3,000	15,830	18,830
Hotel	90,000	116,550	206,550
Maintenance	12,000	20,000	32,000
Van and Petrol	11,200	10,500	21,700
New Construction	45,000	35,000	80,000
Miscellaneous	10,000	0	10,000
**TOTAL**	**299,200**	**351,080**	**650,280**

Pre-multiplying the Leontief inverse matrix by our D vector allows us to obtain the figure of the total economic impact produced by spa tourism. As shown in Table 5, we have obtained a result of **€2,645,313.17**: €1,729,935.98 correspond to direct impact and €915,377.19 to the indirect economic impact. Therefore, spa visitors generate a total multiplier of 1.529.

**Table 5:** Direct, indirect and total impact by sectors (in euros).

			Direct	Indirect	Total
1	A	Agricultural and fishery products	.00	12,112.39	12,112.39
2	B,C,D,E	Industrial products and sanitation	87,500.00	218,777.29	306,277,29
3	F	Building Jobs	80,000.00	79,035.92	159,035.92
4	G,H,I	Commercial, transport and hospitality services	1,317,905.98	257,105.17	1.575,011.15
5	J	Information and communication services	.00	15,997.90	15,997.90
6	K	Financial and insurance services	.00	48,669.25	48,669.25
7	L	Real estate services	.00	100,342.13	100,342.13
8	M,N	Professional, scientific and auxiliary services	227,530.00	173,255.62	400,785.62
9	O,P,Q	Public administration, education and health services	17,000.00	4,887.02	21.887.02
10	R,S,T,U	Artistic, entertainment and other services	.00	5,194.50	5,194.50
		**Total**	**1,729,935.98**	**915,377.19**	**2,645,313.17**

### The Social Accounting Matrix (SAM) and induced effects

The standard IO model proves to be useful to calculate the indirect economic impact brought about by visitors’ spending, since it includes interdependence amongst the production industries of an economy and gives information concerning intermediate and final demand. Nevertheless, something else is needed in order to estimate induced effects, namely macroeconomic accounts. To obtain the relationship between wellness tourism industry and macro-economy, the Catalan IO table is inserted into a SAM. We used the SAM created by Llop [90] for the year 2005 to estimate the induced economic effects. The relations between consumption and income in the Catalan economy are covered. As Table 6 shows, when the calculations include induced effects, there is an increase in the previous economic impact figures, that included only direct and indirect effects. As can be seen in Table 6, the total impact, in terms of output, is €2,961,626.59; whereas in terms of added value it amounts to €1,617,945.90 and in terms of employment 33.16 jobs. All these figures, which have been delimited territorially in order to analyse only the economy of Maresme county, have been calculated too in relative terms. The Value Added impact is .02% of Maresme’s Gross Added Value, the output impact represents .03% of the GDP and the employment impact is .10% of total unemployment. Whereas spa visitors generate a total multiplier of 1.529 taking into account direct and indirect impacts, the multiplier is 1.712 considering also induced effects.

**Table 6:** Total impact of spas.

Concept	Total = Direct+ indirect+ induced
Output	€2,961,626.59
Value Added	€1,617,945.90
Employment	33.16

### Net social value: Cost benefit analysis of investing in spa tourism in Maresme

CBA quantifies in monetary terms both the profit and social costs involved in a specific activity upon the whole society. Next, a socio-economic evaluation based on the CBA, defining and valuing the costs and profits generated by spa tourism in Maresme society is presented.

#### Costs

The year costs of the corresponding spas, provided by their managers, are summarized in Table 4. Total costs of the two spas are estimated to amount to €650,280.

#### Direct economic benefits

We have already estimated that the direct economic benefit of spa tourism amounts to **€1,079,379.00**.

#### Direct health benefits

In this section we will focus on assessing the benefits of the spa’s mineral-medicinal waters on health, and consequently, the savings it generates in social costs.

Clinical studies to identify the percentage of improvement after balneotherapy were searched, being 49% for Arthrosis [95], 22% for Arthritis Rheumatoid [96], 54% for Fibromyalgia [97] and 77.8% for Low Back Pain [98]. Additionally, we have found out the yearly direct cost of this diseases for the Spanish National Health. In regard to Arthrosis, it costs €1,502 per patient per year [99]. The cost per patient per year in the case of Arthritis Rheumtoid amounts to de €5,000 [100] and Fibromyalgia represents an annual cost of €10,000 per patient [101]. Low Back Pain costs annually €8,800 [102]. Whereas 4.37% of the Spanish population suffers from Arthrosis [99], .5% suffers from Arthritis Rheumtoid [100], 2.4% from Fibromyalgia [101] and 54.5% from Low Back Pain [103]. Using the obtained data and taking into account the sum of the number of annual visitors (84) in *Balneari Titus* of Arenys de Mar as well as in the *Balneari Públic de Caldes d’Estrac* that visit the spa during 21 or more days consecutively, the reduction of social costs related to these four diseases by means of using Eq (6) can be found out. The saving for National Health in the expenditures corresponding to the diseases Arthrosis, Rheumatoid Arthritis, Fibromyalgia and Low Back Pain amounts to €327,478.23.

Nevertheless, this amount has been globally estimated, since not only Maresme’s citizens have been surveyed. Therefore, this amount needs to be delimited to the territory of Maresme county. Taking into account that, according to our surveys 30% of visitors are from Maresme, we conclude that the saving in cost for the National Health in Maresme amounts to **€98,243.47**. Nonetheless, we must recognise that these are very conservative hypotheses, given that we are assuming that the users of spas suffer from these diseases in the same proportion than the Spanish population and that only visitors that use a 3-week or longer treatment benefit from balneotherapy.

#### Indirect health benefits

In this section we aim to estimate the indirect benefits of balneotherapy on health. To make this calculation, it must be taken into account that balneotherapy reduces employee absenteeism by 30% [104]. Furthermore, if we take into consideration that the annual average employee absenteeism in Catalonia is 9.9 days, balneotherapy would reduce absenteeism in 2.97 days (30% of 9.9 days). Given that the average annual wage is €23,849 [105], that 41% of the 84 annual customers that use the spa during 21 days or more consecutively spas are active, we conclude that the benefit in euros of balneotherapy on productivity is €6,683.39 (€23,849 x 2.97 / 365 x 41% x 84). Nonetheless, given that only 30% of the users of these spas live in Maresme county, the indirect health benefits amount to €2,005.02 (30% x €6,683.39).

#### Consumers’ benefit

Since the benefit of spa tourism has a market and consequently, a directly observable price, the consumer’s willingness to pay by means of the price they are already paying can be known. This can be related to the consumer surplus theory, understanding this concept as the monetary profit obtained by consumers. In this case, 63.61% of visitors are paying for visiting spas at a price which is lower than what they are willing to pay. It has been estimated that the additional amount of money each person would pay (or the consumer surplus) is: €28.92. By multiplying this value by the 30% of people coming from Maresme and by the total number of annual visitors (3,320), we obtain the value of the spa tourism for the residents is **€28,808.75.**

#### Total benefits

According to Table 7, summing the direct economic benefits, the direct and indirect profits for health and the valuation of spa tourism, the total annual benefit of spa tourism in the Maresme county amounts to **€1,208,436.23**.

**Table 7:** Summary of benefits and costs (in euros).

Benefits	In euros	Costs	In euros
B1. Direct economic impact	1,079,379.00	C1. Expenses and investment	650,280.00
B2. Direct health benefits	98,243.47		
B3. Indirect health benefit	2,005.02		
B4. Consumers’ benefit	28,808.75		
**B. Total**	**1,208,436.23**	**C. Total**	**650,280.00**
**Cost–benefit ratio (B/C)**	**1.858**		
**Net social benefit (B-C)**	**558,156.23**		

### Result of the analysis

Given that the benefits of spa tourism are higher than its costs, according to Table 7, the net social benefits of spa tourism in the Maresme county amount to **€558,156.23**. In particular, by estimating the cost-benefit ratio, we conclude that each euro invested by the Public Administration in spa tourism generates **€1.858** for the Maresme society.

## Discussion

In regard to the motivation of Maresme’s spa users, according to Dimitrovski & Todorović’s [106] clustering, the highest segment of spa users in Maresme can be included in the “relaxation” cluster, whereas using Chen et al.’s [107] typology, the second highest segment can be included in the “physiotherapy group”. Using the clustering performed by Kucukusta & Denizci Guillet [108] for spa users in Hong Kong, most Maresme’s visitors encompass in the “pleasure-oriented” and “health conscious and intellectual” clusters. Finally, using Guo et al.’s [109] segmentation, most Maresme’s visitors corresponds to “treatment oriented spa goers”; conversely, the highest group of this segmentation is “price-sensitive spa goers” and it does not correspond with our visitor’s profile, given that 63.61% of users would pay more money to go to spas. Nonetheless, Guo et al.’s [109] segmentation was carried out for Chinese visitors in Hong Kong’s spas and purchasing power should be taken into account when interpreting these results, whereas Maresme’s visitors are mainly from the same country (Spain, 98%) and the same region (Catalonia, 85%).

On the one hand, IO model results suggest that, taking into account only economic value, investing on spa tourism is a profitable investment. Spas’ activity generates a total multiplier of 1.529 taking into account the direct and indirect effects and 1.712 considering also the induced effects by means of the use of SAM. For the sake of comparison, Zhu [61] concludes that the multiplier in medical tourism on Kwangwon Province is 1.53 whereas Eusébio et al. [10] conclude that the Portuguese Health Tourism Programme for the elderly has a multiplier of 1.654. Therefore, the multipliers obtained for spa tourism are similar, although slightly lower, to those analysed in previous papers in regard to health and medical tourism [10, 61, 79], which ranges between 1.654 and 1.742. Therefore, we can conclude that spa tourism in Maresme is as profitable as medical tourism in other countries, such as Portugal and China.

On the other hand, taking into account the social value generated by spa tourism, direct and indirect health profits have also been considered given that balneotherapy improves several diseases [95–97]. Additionally, consumer’s benefit has also been included. CBA results suggest that investing in spas tourism generates both high net social benefits and cost-benefit ratio; specifically, every euro invested in spa tourism produces 1.858 of return to society. These results have been obtained considering very conservative hypotheses so as to not favour this kind of investment, which could be considered a mischievous practice [110, 111]. The main contribution of CBA to spa tourism, for both practice and policy, is providing decision makers with the information they need to decide about investments, in view that it considers benefits that are not usually taken into account by organisations and governments, who focus almost exclusively on direct economic effects. Furthermore, CBA is also relevant for researchers because it provides a more comprehensive perspective in comparison with analyses that focuses only on economic benefits.

## Conclusions

This article is the first one to analyse both the economic and social yield of investing on spas tourism, describing and monetising different elements that are considered not only economic but also social benefits and costs. This analysis has been conducted in Maresme county, where two spas are located, being one of them public and the other one private. Instead of taking a perspective exclusively based on the traditional IO model, where only economic impact is assessed, social value has also been taken into account by means of a CBA. The latter are less frequently used, given that they require more information and that there is a lack of standardised methodology to assess social value. Nonetheless, CBAs are a more accurate methodology because they take into account social value, such as direct and indirect health benefits. The contribution to the field of this paper is providing economic models to quantify economically direct and indirect health benefits that can be applied, not only to balneotherapy, but also to other kind of therapies.

The results suggest that investing in balneotherapy creates both economic and social value. On the one hand, the economic benefits obtained are 1.712 times the incurred costs–similar to health and medical tourism multipliers [10, 61, 79]–, representing 0.03% of Maresme’s GDO and the employment impact represents 0.10% of total unemployment in the county. On the other hand, investing in balneotherapy creates additional 0.146 euros of social value per euro invested.

There are five policy implications in this article. First, given that there is a lack of standardized methodology, it provides a methodology to quantify the economic and social impact of spa tourism by means of combining direct, indirect and social effects that should be accounted for in order to assess the actual value of these initiatives by local governments. Second, a positive and significant effect generated by the presence of spas in a region in terms of economic and social value is demonstrated by the results of this analysis. Third, spa tourism can be considered another product to be sold by destinations, which should be complemented with other touristic products, namely gastronomic and sporting, so as to create a health tourism package. Fourth, given that the paper performs an evaluation of spa tourism by calculating its economic and social impact, quantitative criteria are given to authorities in order to facilitate the decision making process when choosing which product should be promoted when comparing spa tourism to others options. Finally, Maresme can be considered a successful case and an example to be imitated by other counties that want to change their tourism models.

The limitations of this paper have to do, on the one hand, with the local perspective that has been taken, given that only Maresme county has been analysed. Likewise, most visitors of Maresme spas were older age and retired and this may not be the profile of visitors in other spas, with more young visitors, who differ in terms of habits and purchasing power. For these reasons, future lines of research should focus on performing similar analyses for a wider territory, for instance Catalonia, a region with a considerable number of spas and high-quality thermal water. On the other hand, whereas a limited number of economic and social effects have been considered in this study, social value is a broad term that aims to capture the total net value that an organisation provides to different dimensions in society, such as jobs, benefits for the local community (for instance, opportunities for disadvantaged groups), environmental issues and innovation. For this reason, futures lines of research could also include and assess both costs and returns in terms of CBA analysis of environmental issues (such as reducing waste), innovation, as well as the creation of value for all stakeholders. Additionally, future papers should address the issue of how spa tourism could impact on destination image, being aimed at monetising these effects. For the time being, it has been necessary to ponder over the validity of each and every variable used to monetise social value. Therefore, future papers should create a standardised methodology to assess the social value generated by spas and wellness tourism, which could include, not only the necessary data, but also the key performance indicators to manage these institutions efficiently under a multistakeholders’ perspective. In particular, futures lines of research could use social accounting which allows to quantify social value according to each and every stakeholders’ perspective [35, 36].

## Supporting information

S1 File (ZIP)
