# Supplementary material for: The economic and social value of spa tourism: The case of balneotherapy in Maresme, Spain
Source: PLoS One. 2022 Jan 31;17(1):e0262428. doi: 10.1371/journal.pone.0262428 (PMC8803250; doi:10.1371/journal.pone.0262428)
Supplement: S1 File — (ZIP) [file pone.0262428.s001.zip › 2. Enquesta Caldes.docx]

Data de realització de l’enquesta: ___/___/___

(__) Entre setmana (__) Caps de setmana

Tipus de visitant:

(__) Caldes (__) Fora de Caldes

Dades socioeconòmiques:

1. Edat: _____

2. Gènere: (__) Home (__) Dona

3. Nivell d’estudis:

(__) Obligatòria (__) Secundària post-obligatòria (__) Universitat

4. Procedència:

(__) Catalunya. A quin municipi? __________________

(__) Espanya. A quina província? _________________

(__) Internacional. A quin país? _____________

5. Situació laboral:

(__) Estudiant (__) Desocupat/Inactiu

(__) Jubilat (__) Ocupat

5.1 En el cas d’estar ocupat, contracte:

(__) Indefinit (__) Temporal

(__) Treball autònom

6. Tipus de professió (en el cas d’estar ocupat)

(__) Qualificat (__) No qualificat

6.1 En el cas del treball qualificat:

(__) Administració pública (__) Gerència

(__) Tècnic no gerència

7. Sector:

(__) Primari (__) Construcció

(__) Indústria (__) Serveis

7.1. En cas de treballar en sector serveis:

(__) Comerç (__) Establiments esportius

(__) Turisme (__) Altres serveis

Perfil del visitant / turista:

8. Cada quan va a un balneari?

(__) Primera vegada (__) Setmanalment

(__) Mensualment (__) Esporàdicament

9. Cada quan visita Caldes per a fer l’activitat?

(__) Primera vegada (__) Setmanalment

(__) Mensualment (__) Trimestralment

(__) Esporàdicament

9.1. Quantes vegades anteriorment?

10. Motivació principal de la visita:

(__) Relaxació/Desconnexió (__) Recuperació

(__) Curar una malaltia (__) Prevenció malalties

(__) Oci

10.1. En el cas de motivacions alienes a les anteriors

per efectuar la visita, per què el practiqueu?

(__) Xec regal

(__) Recomanat per altres visitants

(__) Recomanació mèdica

(__) Altres

11. Amb quantes persones ha vingut?

Adults [+ o = 15anys] Nens [< 15 anys]

12. De qui van acompanyats?

(__) Individualment (__) En parella

(__) En família (__) En grup

Perfil de visita:

13. Esteu allotjats al mateix balneari?

(__) Si (__) No

** En el cas de SI estar allotjats, passeu al número 15.

14. Tipus d’allotjament (en cas que NO)

(__) Hotel * (__) Càmping (__) Pensió/Hostal

(__) Hotel ** (__) Apartaments

(__) Hotel *** (__) Alberg (__) Segona residència

(__) Hotel **** (__) Casa familiars o amics

(__) Altres Quin?____________________________

15. Número de nits allotjats:

16. Règim d’allotjament:

(__) Dormir (__) Dormir i esmorzar (__) MP (__) PC

17. Quin és el principal motiu del seu allotjament a la destinació?

(__) Qualitat-preu (__) Desconeixement d’alternatives

(__) Proximitat de la platja (__) Gastronomia

(__) Cultura (__) Esport (__) Altres. Quin?________

18. Ha vingut al balneari via IMSERSO?

Nivell de despesa:

19. Despesa en allotjament:

20. Despesa en tractaments:

21. Despesa global:

| Despesa global | Sí | NO | € | % |
| --- | --- | --- | --- | --- |
| Restaurant |  |  |  |  |
| Manutenció |  |  |  |  |
| Compres |  |  |  |  |
| Cultura |  |  |  |  |
| Esport |  |  |  |  |

Disponibilitat a pagar:

22. Si la despesa que acaba de calcular augmentés, encara

hagués decidit venir-hi?

(__) Si (__) No (final qüestionari)

22.1. Quants euros extres estaria disposat a pagar?

30

60

90

120

150

180

210
