# Supplementary material for: The economic and social value of spa tourism: The case of balneotherapy in Maresme, Spain
Source: PLoS One. 2022 Jan 31;17(1):e0262428. doi: 10.1371/journal.pone.0262428 (PMC8803250; doi:10.1371/journal.pone.0262428)
Supplement: S1 File — (ZIP) [file pone.0262428.s001.zip › 2. Survey Caldes.docx]

Socioeconomic data:

1. Age:

2. Gender: (__) Male (__) Female

3. Educational attainment:

(__) Compulsory (__) Post-compulsory Secondary

(__) University

4. Origin:

(__) Catalonia Which municipality?

(__) Spain Which province?

(__) International Which country?

5. Employment status:

(__) Student (__) Unemployed/Inactive

(__) Retired (__) Active

5.1 In case of being employee, contract:

(__) Indefinite (__) Temporary

(__) Autonomous worker

6. Type of profession (in case of being active)

(__) Qualified (__) Non-qualified

6.1 If qualified job:

(__) Public Administration (__) Management

(__) Non-management specialists

7. Sector:

(__) Primary (__) Construction

(__) Industry (__) Services

7.1. If working in the service sector:

(__) Commerce (__) Sports facilities

(__) Tourism (__) Other services

Visitor/tourist’s profile:

Date of the survey: ___/___/___

(__) Weekdays (__) Weekends

Type of visitor:

(__) Caldes (__) Outside Caldes

8. How often does visit a spa?

(__) First time (__) Weekly

(__) Monthly (__) Sporadically

9. How often does visit Caldes for the activity?

(__) First time (__) Weekly

(__) Monthly (__) Quarterly

(__) Sporadically

9.1. How many times previously?

10. Main motivation for the visit:

(__) Relax/Disconnect (__) Recovery

(__) Healing a sickness (__) Preventing a sickness

(__) Leisure

10.1. In case of different reasons for the visit,

why are you doing it?

(__) Gift voucher

(__) Recommended by other visitors

(__) Medical advice

(__) Other

11. How many people are accompanying you?

Adults [+ o = 15anys] Children [< 15 anys]

12. Who is accompanying you?

(__) Individually (__) As a couple

(__) As a family (__) In a group

Visit’s profile:

13. Are you staying in your usual residence?

(__) YES (__) NO

** If YES, go to number 15.

14. Type of accomodation (if the answer was NO)

(__) Hotel * (__) Camping (__) Guesthouse

(__) Hotel ** (__) Apartments (__) Vacation home

(__) Hotel *** (__) Hostel

(__) Hotel **** (__) Friends’ or relatives’ home

(__) Other ____________________________

15. Number of nights:

16. Accommodation type:

(__) Self-catering (__) Bed and Breakfast (__) HB (__) FB

17. What the main reason for your accommodation at the destination?

(__) Price/quality (__) Unawareness of alternatives

(__) Proximity to the beach (__) Gastronomy

(__) Culture (__) Sport (__) Other _______

18. Are you visiting the spa through IMSERSO?

Level of spending:

19. Spending in accommodation:

20. Spending in treatments:

21. Overall spending:

| Overall spending | YES | NO | € | % |
| --- | --- | --- | --- | --- |
| Restaurant |  |  |  |  |
| Provisions |  |  |  |  |
| Shopping |  |  |  |  |
| Culture |  |  |  |  |
| Sports |  |  |  |  |

Willingness to spend:

22. Should the expenditure you have estimated increase, would you still come?

(__) Yes (__) No (end of questionnaire)

22.1. How many extra € would you be willing to spend?

30

60

90

120

150

180

210
